# ASSOCIATION OF RELIGIOUS ATTENDANCE WITH NEUROPSYCHIATRIC SYMPTOMS, COGNITION, AND SLEEP IN COGNITIVE IMPAIRMENT

**DOI:** 10.1093/geroni/igac059.2946

**Published:** 2022-12-20

**Authors:** Katherine Britt, Kathy Richards, Shelli Kesler, Gayle Acton, Jill Hamilton, Kavita Radhakrishnan

**Affiliations:** University of Pennsylvania, Austin, Texas, United States; University of Texas at Austin, The, Austin, Texas, United States; University of Texas at Austin, The, Austin, Texas, United States; University of Texas at Austin, The, Austin, Texas, United States; Emory University, Atlanta, Georgia, United States; University of Texas at Austin, The, Austin, Texas, United States

## Abstract

Neuropsychiatric symptoms (NPS), cognitive decline, and sleep disturbances are common among older adults with cognitive impairment. Religious practices may protect mental and physical health, yet few studies have been reported in older adults with cognitive impairment. Utilizing the Health and Retirement Study in 2006 and 2008 and sub study, Aging, Demographics, and Memory study in 2006–2007 and 2008–2009, we examined the association of religious attendance with NPS, cognition, and sleep disturbances controlling for social interaction in older adults with cognitive impairment (N = 63). Bootstrapped Spearman’s partial Rho correlation was conducted separately for time points one (T1) and two (T2); Wilcoxon signed-rank tests were used to examine significant change over time. Mean age was 81.89(5.26) years, 65.9% were non-Hispanic White, 50.1% were female, and mean cognition (Clinical Dementia Rating) was .94(.228). Significant changes over 1.5 years were found for sleep disturbances but not for NPS and cognition. Significant associations were found for religious attendance and NPS (T1: rs (97)= - .103, 95% CI [-.108, -.098], p < .0005 and T2: -.243, 95% CI [-.246,-.239], p < .0005), cognition, (T1: rs (97) = - .119, 95% CI [-.122, -.115], p < .0005, and T2: rs (97) = -.104, 95% CI [-.107,-.102], p < .0005), and sleep disturbances, (T1: rs (97) = .028, 95% CI [.023, .033], p < .001, and T2: rs (97) = -.051, 95% CI [-.056,-.047], p < .001). Increased religious attendance was associated with lower NPS and cognition at both time points and greater sleep disturbances at T1 but lower at T2. Longitudinal studies are needed to examine associations further.

